# Evaluation of TGF-β1 and EGFR in Cleft Affected Lip Mucosa

**DOI:** 10.15388/Amed.2021.28.1.14

**Published:** 2021-03-25

**Authors:** Olga Rimdenoka, Māra Pilmane

**Affiliations:** Riga Stradins University, Institute of Anatomy and Anthropology; Riga Stradins University, Institute of Anatomy and Anthropology

**Keywords:** orofacial cleft, lip mucosa, TGF-β1, EGFR

## Abstract

**Background.:**

The morphopathogenesis of orofacial cleft development is only partly understood; therefore, it is important to identify factors, which possibly could be involved in it. The aim of the study was to evaluate the distribution of TGF-β1 and EGFR-containing cells in cleft affected lip mucosa.

**Materials and Methods.:**

The study group included lip mucosa tissue samples from 14 patients with orofacial cleft. The control group contained 11 healthy oral mucosa tissue samples. The tissue sections were stained by immunohistochemistry for TGF-β1 and EGFR. The expression of positive structures was graded semiquantitatively. IBM SPSS 26.0 was used for statistical analysis, Spearman`s rank correlation and Mann-Whitney U tests were performed.

**Results.:**

Mostly few to moderate number (+/++) of TGF-β1-containing cells was found in epithelium, also the same number of fibroblasts and macrophages was seen in the *lamina propria* of cleft affected lip mucosa. Meanwhile, healthy oral mucosa on average demonstrated a moderate number (++) of TGF-β1-containing epithelial cells, fibroblasts, and macrophages. A variable, mostly indistinct number of EGFR-containing cells was seen in the epithelium of cleft affected lip mucosa, meanwhile, mostly no (0) EGFR positive cells were found in the epithelium of healthy mucosa. Statistically significantly less TGF-β1-containing cells were found in the epithelium of cleft affected lip mucosa than in the healthy mucosa (U=33.000; p=0.015). Also, the *lamina propria* of cleft affected lip mucosa showed statistically significantly less TGF-β1 immunoreactive fibroblasts and macrophages than the healthy mucosa (U=28.500; p=0.006).

**Conclusions.:**

The decreased number of TGF-β1-containing epithelial cells, fibroblasts and macrophages in cleft affected lip mucosa proves the role of problematic tissue remodelation in the cleft pathogenesis. The distribution of EGFR in cleft affected and healthy mucosa is similar and possibly does not play a role in the cleft development of humans.

## Abstract (lt)

**Transformuojančio augimo veiksnio beta 1 ir epidermio augimo veiksnio receptoriaus įvertinimas gleivinėje esant lūpos nesuaugimams:**

**Santrauka. Apžvalga.**

Lūpos nesuaugimo išsivystymo morfopatogenezė nėra iki galo aiški, todėl labai svarbu nustatyti tam turinčius įtakos veiksnius. Tyrimo tikslas – įvertinti ląstelių turinčių TGF-1 ir EGFR pasiskirstymą nesuaugusių lūpų gleivinėje.

**Metodai.**

Tyrėjai paėmė lūpų gleivinės audinių mėginių iš 14 pacientų, turinčių nesuaugusias lūpas. Kontrolės grupė – 11 sveikos burnos gleivinės audinių pavyzdžių. Audinių dalys TGF-β1 ir EGFR buvo nudažytos imunohistocheminiu dažymo metodu. Teigiamų struktūrų raiška buvo vertinta pusiau kiekybiniu būdu. IBM SPSS 26.0 buvo naudojama statistinei analizei atlikti, taip pat atlikta Spirmeno ranginė koreliacija bei *Mann-Whitney* U testai.

**Rezultatai.**

Epitelyje buvo aptiktas nedidelis TGF-β1 turinčių ląstelių skaičius (+/++), toks pats skaičius fibrobalstų ir makrofagų buvo rastas nesuaugusių lūpų gleivinės jungiamojo audinio sluoksnyje. Sveikos burnos gleivinėje buvo vidutiniškai nedidelis kiekis (++) TGF-β1 turinčių epitelinių ląstelių, fibroblastų ir makrofagų. Kintantis, dažniausiai nedidelis EGFR turinčių ląstelių skaičius buvo nustatytas nesuaugusių lūpų gleivinių epitelyje, o jokių (0) EGFR teigiamų ląstelių nebuvo aptikta sveikos gleivinės epitelyje. Statistiškai gerokai mažiau TGF-β1 turinčių ląstelių buvo aptikta nesuaugusių lūpų gleivinės epitelyje negu sveikoje gleivinėje (U = 33 000; p = 0,015). Be to, nesuaugusių lūpų gleivinės jungiamojo audinio sluoksnyje buvo statistiškai gerokai mažiau TGF-β1 imunoreaktyvių fibroblastų ir makrofagų negu sveikoje gleivinėje (U = 28 500; p = 0,006).

**Išvados.**

Sumažėjęs**** TGF-β1 turinčių epitelinių ląstelių, fibroblastų ir makrofagų skaičius nesuaugusių lūpų gleivinėje rodo probleminio audinio remodeliacijos įtaką nesuaugimo patogenezėje. EGFR paplitimas nesuaugusioje ir sveikoje gleivinėje yra panašus ir galimai neturi įtakos žmogaus nesuaugusios lūpos išsivystymui.

**Reikšminiai žodžiai:** lūpų nesuaugimas, burnos gleivinė, TGF-β1, EGFR.

## Introduction

Orofacial clefts are common congenital malformations of craniofacial region, which are inherited in a multifactorial model, where genetic and environmental risk factors interact. The prevalence of nonsyndromic cleft lip with or without cleft palate is approximately 1 in 300–2500 depending on the population [1]. Despite the high prevalence, the morphopathogenesis of orofacial cleft development is only partly understood; therefore, it is important to identify the factors, which possibly could be involved in it. Two of these possible factors, which could possibly play a role in the development of orofacial clefts, are transforming growth factor beta 1 (TGF-β1) and epidermal growth factor receptor (EGFR).

TGF-β1 is a member of TGF-β family, which achieves its cellular effects via binding to serine/threonine kinase receptors [2]. It has been implicated in embryogenesis, maintenance of tissue homeostasis and wound healing [3,4]. It regulates cell proliferation and extracellular matrix synthesis and increases the process of tissue remodelation by inducing biosynthesis of collagens I and III, laminin and fibronectin [5,6]. TGF-β1 is present in both normal tissue and transformed cells [7]. Different studies describe the involvement of TGF-β1 in pathogenesis of various diseases such as endometriosis, hepatocellular carcinoma, idiopathic pulmonary fibrosis, and cleft palate [8,9,10,11]. It is found that TGF-β1 plays an essential role in all phases of palate development, therefore abnormal expression of TGF-β1 can lead to pathological development of palate [4,12,13]. As TGF-β1 is one of the strongest growth factors, which provides and indicates the previously mentioned tissue remodelation processes and wound healing [3,14,15], it is essential to detect this factor in cleft affected lip to get an insight into these processes in the context of cleft lip morphopathogenesis. Now there are no previous studies which describe quantitative expression of TGF-β1 positive structures in cleft affected lip tissue.

EGFR is a type I receptor tyrosine kinase. Along with its ligands, EGFR takes part in regulation of multiple cellular pathways [16]. It plays a significant role in several cellular processes such as cellular proliferation, differentiation, migration, and apoptosis [17]. EGFR is contributed to normal and neoplastic growth processes in humans. Increased EGFR activity has been detected in a variety of tumours such as glioblastoma, nonsmall cell lung cancer, head and neck, breast, colorectal, ovarian, prostate, and pancreatic cancers. The activity can be increased due to higher epidermal growth factor synthesis, EGFR overexpression or EGFR mutation [18]. Studies show that EGFR signalling is necessary for normal craniofacial development and palate closure, which gives an evidence that decreased activity of the receptor may lead to impaired craniofacial development and cleft formation [19,20]. EGFR is also one of the most important growth factors receptors, stimulating tissue growth and regeneration processes, and in possible conjunction with another strongest growth factor – TGF-β1 – is being particularly interesting in cleft patients [14,17,21,22]. At the moment there are no studies which describe the quantitative expression of EGFR positive structures in cleft affected lip tissue.

On the basis of above mentioned the aim of the study was to evaluate the distribution of TGF-β1 and EGFR-containing cells in cleft affected lip mucosa.

## Materials and methods

Oral mucosa tissue samples from 25 patients were used in the study. The tissue samples were divided in two groups. The study group included 14 lip mucosa tissue samples from 3 months to 1 year and 1 month old children (5 girls and 9 boys). Two children had bilateral orofacial cleft; meanwhile 12 children were diagnosed with unilateral orofacial cleft. Orofacial clefts were nonsyndromic in all patients. The tissue samples were obtained during lip correction surgery in the Cleft Lip and Palate Centre at the Institute of Stomatology of Riga Stradins University (Table 1). The control group included 11 healthy oral mucosa tissue samples from 6 years and 9 months to 14 years and 5 months old children (6 girls and 5 boys). All 11 control group tissue samples were stained for TGF-β1, and 6 control group tissue samples were stained for EGFR. The control group tissue samples were obtained during tooth extraction, opening of an embedded tooth, benign tumour removal or surgical treatment of traumatic injury (Table 2).

**Table 1.** Information about the study group patients.

**Patient**	**Sex**	**Age**	**Diagnosis**
1	Boy	3 months	*Cheilognathouranoschisis dextra*
2	Boy	3 months	*Cheilognathouranoschisis bilateralis*
3	Boy	3 months	*Cheilognathouranoschisis sinistra*
4	Boy	4 months	*Cheilognathouranoschisis dextra*
5	Boy	1 year and 1 month	*Cheilognathouranoschisis bilateralis*
6	Boy	4 months	*Cheilognathouranoschisis sinistra*
7	Boy	3 months	*Cheilognathouranoschisis dextra*
8	Boy	5 months	*Cheilognathouranoschisis sinistra*
9	Girl	6 months	*Cheilognathouranoschisis sinistra*
10	Girl	4 months	*Cheilognathouranoschisis dextra*
11	Girl	4 months	*Cheilognathouranoschisis sinistra*
12	Boy	4 months	*Cheilognathouranoschisis sinistra*
13	Girl	3 months	*Cheilognathouranoschisis sinistra*
14	Girl	3 months	*Cheilognathouranoschisis sinistra*

**Table 2.** Information about the control group patients.

**Patient**	**Sex**	**Age**	**Operation, during which the tissue samples were obtained**
1	Girl	13 years and 8 months	Tooth extraction
2	Girl	14 years and 5 months	Opening of an embedded tooth
3	Boy	10 years and 2 months	Tooth extraction
4	Boy	8 years and 2 months	Tooth extraction
5	Girl	9 years and 9 months	Tooth extraction
6	Girl	6 years and 9 months	Tooth extraction
7	Boy	8 years and 9 months	Tooth extraction
8	Girl	10 years and 5 months	Tooth extraction
9	Boy	11 years and 7 months	Tooth extraction
10	Boy	11 years and 8 months	Surgical correction of a traumatic injury
11	Girl	12 years and 8 months	Removal surgery of a benign tumour

The tissue samples were obtained during surgical intervention after written informed consent from the parents of all 25 patients was received. The study was performed in accordance with the 1964 Declaration of Helsinki. The study was approved by the Ethical Committee at Riga Stradins University (22.05.2003; 17.012013; 5/25.06.2018).

All tissue samples were fixed in Stefanini`s solution, which was made of 20 g paraformaldehyde, 150 ml picric acid, 425 ml Sorensen’s phosphate buffer (pH 7.2) and 425 ml distilled water [23]. After that, the samples went through the dehydration and were embedded in paraffin.

TGF-β1 (code orb77216, polyclonal antibody, dilution 1:100, Biorbyt, Great Britain) and EGFR (sc-71034, mouse monoclonal antibody, dilution 1:100, Santa Cruz, USA) primary antibodies were used by biotin–streptavidin immunohistochemistry (IMH) [24]. Further, all tissue samples were counterstained with haematoxylin to stain the nuclei blue. All antibodies, which were used in the study, were tested for positive and negative control.

The number of immunoreactive structures was graded semiquantitatively. Respectively, no positive structures in the visual field were labelled as 0; occasional positive structures in the visual field were labelled with 0/+; few positive structures in the visual field were labelled with +; few to moderate number of positive structures in the visual field was labelled with +/++; moderate number of positive structures in the visual field was labelled with ++; moderate to numerous positive structures in the visual field were labelled with ++/+++; numerous positive structures in the visual field were labelled with +++; numerous to abundant positive structures in the visual field were labelled with +++/++++; and abundance of positive structures in the visual field was labelled with ++++ [25].

SPSS software, version 26.0 (IBM Company, Chicago, IL, USA) was used for statistical analysis. Spearman`s rank correlation coefficient was calculated, where r = 0-0.2 was assumed as a very weak correlation, r = 0.2-0.4 – a weak correlation, r = 0.4-0.6 – a moderate correlation, r = 0.6-0.8 – a strong correlation, and r = 0.8-1.0 – a very strong correlation. Mann-Whitney U test was performed to compare the study group and the control group. The level of significance for both tests was chosen as 5% (p-value < 0.05).

Leica DC 300F digital camera was used for visual illustration. Image processing and analysis was performed by Image Pro Plus software (Media Cybernetics, Inc., Rockville, MD, USA).

## Results

**Figure 1.**
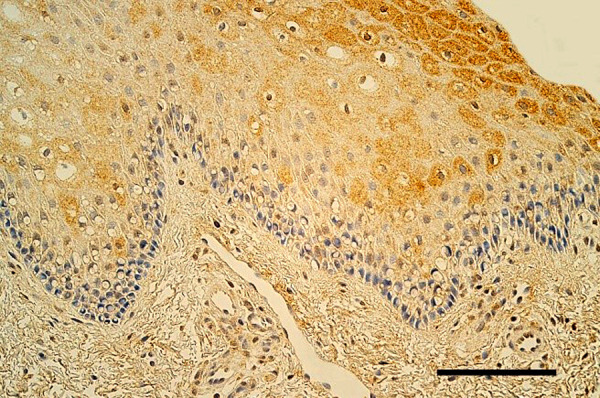
Few to moderate number of TGF-β1-containing cells in the epithelium and *lamina propria* of lip in 5 months old boy with unilateral orofacial cleft. TGF-β1 IMH, 250x. Scale bar 32 µm.

**Figure 2.**
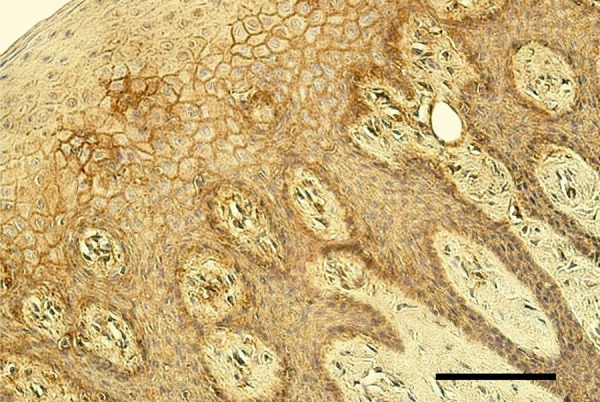
Moderate number of TGF-β1-containing cells in the oral mucosa of healthy 8 years and 2 months old boy. TGF-β1 IMH, 200x. Scale bar 40 µm.

The epithelium demonstrated a variable number of TGF-β1-containing cells, but mostly few to moderate number (+/++) of cells was seen in the cleft affected lip mucosa (Figure 1). The distribution of TGF-β1 immunoreactive fibroblasts and macrophages was similar also in *lamina propria* of the patients. On average a moderate number (++) of TGF-β1 positive cells was seen in the healthy oral mucosa (Figure 2). The tissue samples showed from few (+) to numerous (+++) TGF-β1-containg fibroblasts, macrophages, and epithelial cells.

**Figure 3.**
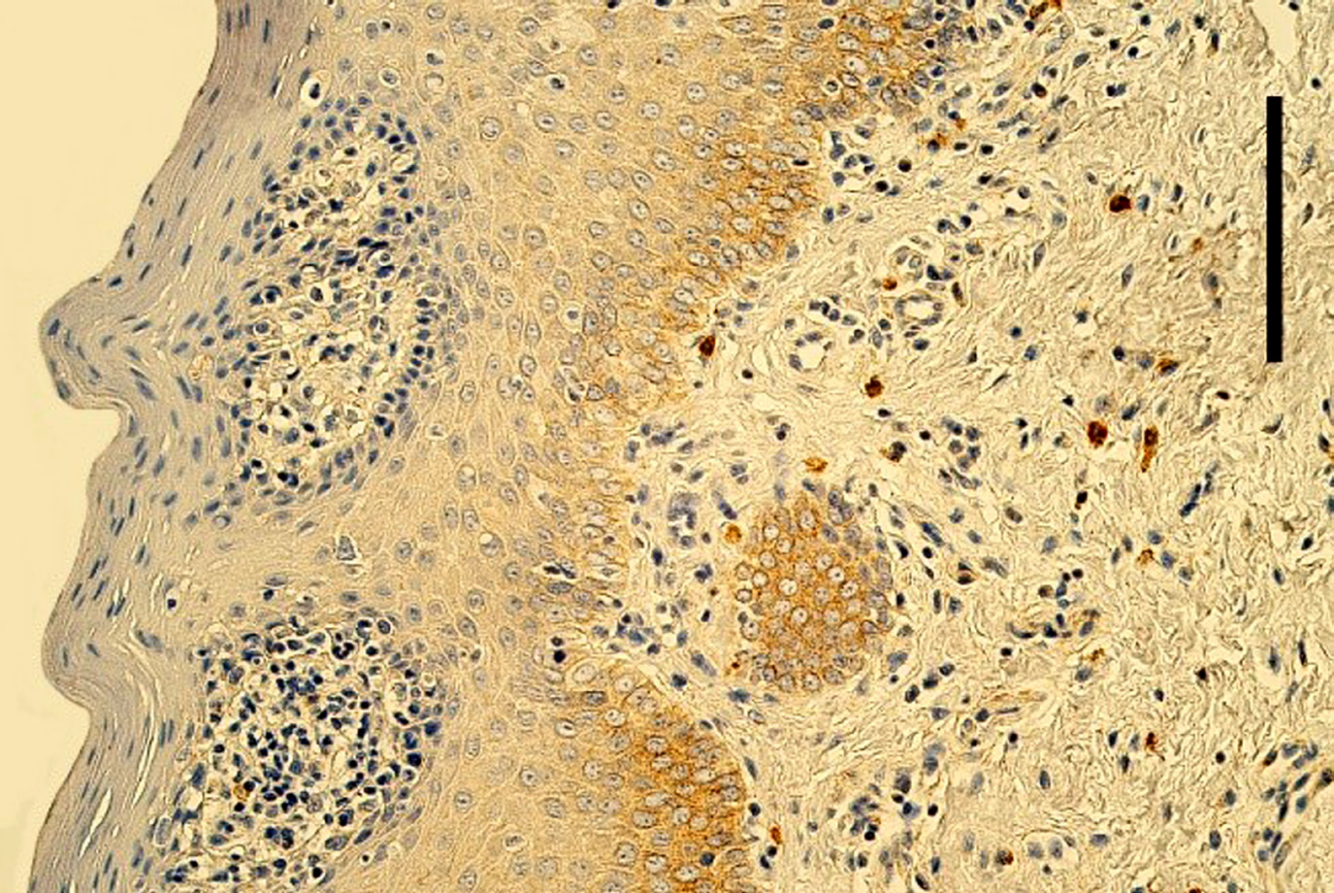
Few EGFR positive cells in the epithelium of lip mucosa in 4 months old girl with unilateral orofacial cleft. EGFR IMH, 250x. Scale bar 32 µm.

**Figure 4.**
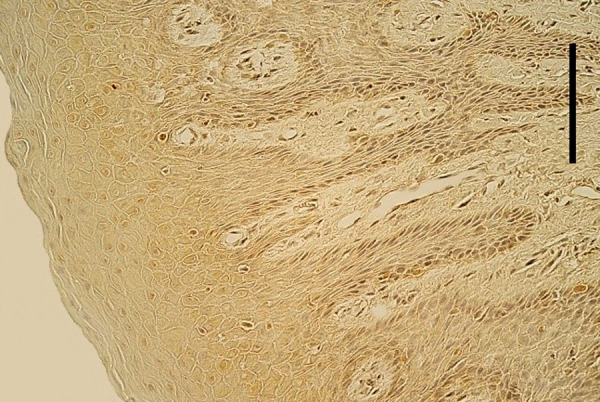
Almost the lack of EGFR-containing cells in the oral mucosa of healthy 8 years and 2 months old boy. EGFR IMH, 250x. Scale bar 32 µm.

Mostly an indistinct number (0/+) of EGFR-containing cells was seen in the epithelium of cleft affected lip mucosa, while one tissue sample demonstrated a moderate number (++) and one – up to numerous (++/+++) EGFR-containing cells (Table 3, Figure 3). Meanwhile, mostly no (0) EGFR-containing cells were found in the epithelium of healthy oral mucosa (Figure 4). Only two healthy mucosa samples demonstrated occasional (0/+) and few (+) EGFR immunoreactive epithelial cells (Table 4).

Statistically significantly less TGF-β1-containing cells were found in the epithelium of cleft affected lip mucosa than in the healthy mucosa (U=33.000; p=0.015). Also, the *lamina propria* of cleft affected lip mucosa showed statistically significantly less TGF-β1 immunoreactive fibroblasts and macrophages than the healthy mucosa (U=28.500; p=0.006). No statistically significant correlation was detected between the number of EGFR and TGF-β1 positive structures in the cleft affected lip mucosa.

**Table 3.** The number of immunoreactive cells in the cleft affected lip mucosa.

Patient	TGF-β1		EGFR	
	Epithelium	*Lamina propria*	Epithelium
1	++	+/++	+/++
2	++	+	+
3	+/++	+/++	0
4	+/++	+/++	0/+
5	0/+	0/+	0
6	+/++	+/++	+/++
7	+/++	++	++
8	+/++	+/++	+/++
9	+/++	+	0
10	0/+	0	0
11	+	+/++	0/+
12	+/++	++	0/+
13	+/++	0/+	+
14	+/++	+/++	++/+++
**On average**	+/++	+/++	0/+ to +

Abbreviations: TGF-β1 – transforming growth factor beta 1; EGFR – epidermal growth factor receptor; 0 – no positive structures, 0/+ – occasional positive structures, + – few positive structures, +/++ – few to moderate number of positive structures, ++ – moderate number of positive structures, ++/+++ – moderate number to numerous positive structures in the visual field.

**Table 4.** The number of immunoreactive cells in the healthy oral mucosa.

Patient	**TGF-β1**		**EGFR**	
	Epithelium	*Lamina propria*	Epithelium
1	+/++	+/++	0
2	+	+	0
3	++/+++	++/+++	0
4	++/+++	++/+++	0
5	+/++	+/++	+
6	++	++	0/+
7	+++	+++	**On average 0**
8	++/+++	++/+++	
9	++	++	
10	+++	+++	
11	+/++	+/++	
**On average**	++	++	

Abbreviations: TGF-β1 – transforming growth factor beta 1; EGFR – epidermal growth factor receptor; 0 – no positive structures, 0/+ – occasional positive structures, + – few positive structures, +/++ – few to moderate number of positive structures, ++ – moderate number of positive structures, ++/+++ – moderate number to numerous positive structures, +++ – numerous positive structures in the visual field.

## Discussion

Orofacial cleft is a pathology with frequent occurrence and extensive psychological, surgical, speech and dental involvement, which emphasizes the importance of understanding the underlying causes of it [13]. Therefore, it is important to identify different tissue factors, including TGF-β1 and EGFR, which possibly could be involved in the development of orofacial cleft.

The age of the patients in the study group varied from 3 months to 1 year and 1 month, meanwhile in the control group it was from 6 years and 9 months to 14 years 5 months. The possibilities to obtain tissue samples from so small and healthy children for the control group were very limited due to ethical reasons. Therefore, in the control group were included tissue samples, obtained from children, who underwent surgical manipulations inside the oral cavity. As the oral cavity development is finished after the complete permanent teeth eruption, which was not seen yet in our research groups, it was possible to compare these groups despite the age difference.

The tissue samples of the study group were obtained from lip, meanwhile the control group contained samples from different regions of oral cavity. The ethical reasons restricted possibilities to obtain tissue exactly from lip mucosa in controls, and surgical intervention in lip is quite rare procedure during so early childhood. Thus, the control group was formed from the patients, who requested surgical manipulations in various regions of oral cavity. As our controls demonstrated similar results in appearance of TGF-β1 and EGFR, we suggest also the similar common expression of them in oral cavity, what gives a possibility to compare the factor distribution between the controls and the patients even despite the different developmental sources of oral cavity (different developmental primordia of tissue). The difference in appearance of TGF-β1 in our patients seemingly connects to the disordered TGF-β1 pathway in cleft affected tissue, as some authors also have not seen the difference in expression of TGF-β1 in different regions of oral cavity [26].

Only few to moderate number of TGF-β1-containing cells was detected in cleft affected lip mucosa. Meanwhile, a moderate number of TGF-β1 immunoreactive epithelial cells, fibroblasts and macrophages was detected in healthy oral mucosa. Statistically significantly less TGF-β1-containing cells were found in cleft affected lip mucosa than in the healthy mucosa, which confirms that expression of TGF-β1 is decreased in cleft affected mucosa, and thus we suggest that the molecular processes promoted and performed by the factor, are impaired in cleft tissue. TGF-β1 plays a role in tissue remodelation, cell proliferation, apoptosis, cell migration and extracellular matrix synthesis and deposition [27] what are crucial for orofacial region development. The impairment of them can lead to cleft lip formation, and also to the extensive tissue remodelation in postnatal life. Our data show indirectly the same as the studies performed on mice about the loss of TGF-β1 signalling in palatal epithelium in cleft soft palate with the following reduced proliferation of palatal mesenchymal cells [28]. However, there are also some contrary data developed in small population in India showing, that genetic polymorphism of TGF-β1 does not predict the development of nonsyndromic cleft [29], and another study in Central European population showed that TGF-β1 receptor might be not associated with the development of orofacial cleft [30]. So, it can be suggested that TGF-β1 pathway is important, but not probably the only player in the pathogenesis of orofacial clefts, and larger population research is required to clarify the real role of TGF-β1 in clefts.

A variable and mostly indistinct number of EGFR-containing cells was seen in the epithelium of cleft affected lip mucosa, meanwhile healthy oral mucosa mostly demonstrated no EGFR positive epithelial cells. There was no statistically significant difference between the patients and the controls. These data respond to the study in Italian population reporting about the lack of link between EGFR polymorphism and orofacial cleft development in humans [31]. However, some previous studies have described that EGFR might be involved in cleft palate development in mice due to the fact, that EGFR-deficient mice have very high incidence of cleft palate [19]. Also, the other study from China found out that single nucleotide polymorphism in EGFR associated gene is related to nonsyndromic orofacial cleft development [20]. According to the diverse results of the studies and our results, it can be suggested that the variability of EGFR expression in cleft affected tissue may give evidence about different pathogenetic pathways of orofacial cleft development between the species, with the decreased expression of EGFR in animals.

Beside EGFR and TGF-β1, also the role of other tissue factors like growth factors and their receptors, which are involved in tissue remodelation processes, has been studied. Fibroblast growth factor receptor 1 (FGFR1) and TGF-β1 have been already reported to be the main tissue stimulating growth factors in cleft affected palate tissue [32]. Another study reported a scarce expression of TGF-β3 in cleft affected tissue, suggesting that this factor might be involved in cleft morphopathogenesis [33]. Expression of bone morphogenetic proteins 2 and 4 (BMP-2/4) was found to be probably delayed in cleft palate disordered soft tissue, but still was proliferation, differentiation, and tissue, especially, bone remodelation signal [34]. Another study showed that bone morphogenetic protein 7 (BMP-7) deficient mice develop the lack of palatal fusion [35]. Meanwhile, more numerous nerve growth factor receptor (NGFR) positive structures have been associated with orofacial clefts before mixed dentition [32].

Studies also have described the importance of tissue degradation factors like matrix metalloproteinases (MMPs) and their inhibitors in pathogenesis of clefts. The increased expression of MMP-2 and tissue inhibitor of metalloproteinase 2 (TIMP-2) was found to be implicated in the regulation of cell migration during extracellular matrix turnover in unilateral and bilateral orofacial clefts, meanwhile increased expression of MMP-9 and TIMP-4 was found to be significant in regulation of remodelation, especially, in unilateral orofacial clefts [36].

Also, primordia of neuronal and endothelial origin cells were studied in cleft affected tissue. So, high expression of nestin has been found, indicating a potentially increased processes of tissue regeneration; meanwhile, CD34 positive cells suggested increased angiogenesis in cleft affected oral mucosa [33]. Interestingly, the involvement of various gene products revealed higher number of BARX homeobox 1 (BARX1) gene protein-containing structures in cleft affected tissue of mixed dentition age [32], while mostly indistinct expression of Msh homeobox 1 (MSX1), paired box 9 (PAX9), receptor like tyrosine kinase (RYK), and interferon regulatory factor 6 (IRF6) gene proteins has been reported in bone of cleft affected palate, what might indicate incomplete cellular differentiation, proliferation, and migration in cleft disordered hard tissue [37].

Also, expression of various interleukins (ILs) in cleft affected tissue has been researched. Epithelium has been found to be the main source of interleukin expression in cleft affected tissue. Rich and similar expression of IL-1 and IL-10 has been found, suggesting the balance between pro-inflammatory and anti-inflammatory tissue response with possibly dysregulated tissue homeostasis, what was indicated by increased expression of IL-8. The study also suggested self-protection compensatory mechanism for intensification of local inflammatory immune response provided by IL-1, IL-4, IL-8, IL-10 without involvement of all pro-inflammatory cytokines (IL-6 was not involved). Additionally, possible involvement of IL-8 and IL-10 in cellular proliferation because of correlation with the expression of Ki67 has been demonstrated. The same study suggested that intensification of local tissue immune response in cleft affected tissue might be regulated by the main pro-inflammatory cytokine – IL-1 [38]. Significant expression of IL-7, β-defensin 2 and IL-10 was seen in cleft affected hard tissue, what proved prominent immune response in cleft affected hard tissue [37].

The all above mentioned suggest the real multifactorial morphopathogenesis of cleft lip and palate, which more likely requires very personalised access to research in this field.

## Conclusions

The decreased number of TGF-β1-containing epithelial cells, fibroblasts and macrophages in cleft affected lip mucosa proves the role of problematic tissue remodelation in the cleft pathogenesis. The distribution of EGFR in cleft affected and healthy mucosa is similar and possibly does not play a role in the cleft development of humans.

## References

[ref1] Kempa I., Ambrozaitytė L., Stavusis J., Akota A., Barkane B., Krumina A., Matulevičienė A., Utkus A., Kučinskas V., Lace B. (2014). Association of BMP4 polymorphisms with non-syndromic cleft lip with or without cleft palate and isolated cleft palate in Latvian and Lithuanian populations. Stomatologija. 16 (3): 94-101. https://sbdmj.lsmuni.lt/143/143-03.pdf 25471993

[ref2] Massagué J., Seoane J., Wotton D. (2005). Smad transcription factors. Genes Dev. 19 (23): 2783-810. DOI: 10.1101/gad.1350705 16322555

[ref3] Roberts C.J., Birkenmeier T.M., McQuillan J.J., Akiyama S.K., Yamada S.S., Chen W.T., Yamada K.M., McDonald J.A. (1988). Transforming growth factor beta stimulates the expression of fibronectin and of both subunits of the human fibronectin receptor by cultured human lung fibroblasts. Biol Chem. 263 (10): 4586-92. https://www.jbc.org/content/263/10/4586.long 2965146

[ref4] Heldin C.H., Landström M., Moustakas A. (2009). Mechanism of TGF-beta signaling to growth arrest, apoptosis, and epithelial-mesenchymal transition. Curr Opin Cell Biol. 21 (2): 166-76. DOI: 10.1016/j.ceb.2009.01.021 19237272

[ref5] Gehris A.L., D’Angelo M., Greene R.M. (1991). Immunodetection of the transforming growth factors beta 1 and beta 2 in the developing murine palate. Int J Dev Biol. 35 (1): 17-24. http://www.ijdb.ehu.es/web/paper. php?doi=1714290 1714290

[ref6] Casini A., Pinzani M., Milani S., Grappone C., Galli G., Jezequel A.M., Schuppan D., Rotella C.M., Surrenti C. (1993). Regulation of extracellular matrix synthesis by transforming growth factor beta 1 in human fat-storing cells. Gastroenterology. 105 (1): 245-53. DOI: 10.1016/0016-5085(93)90033-9 8514041

[ref7] Roberts A.B., Anzano M.A., Lamb L.C., Smith J.M., Sporn M.B. (1981). New class of transforming growth factors potentiated by epidermal growth factor: isolation from non-neoplastic tissues. Proc Natl Acad Sci USA. 78 (9): 5339-43. DOI: 10.1073/pnas.78.9.5339 6975480 PMC348740

[ref8] Zhang J., Li H., Yi D., Lai C., Wang H., Zou W., Cao B. (2019). Knockdown of vascular cell adhesion molecule 1 impedes transforming growth factor beta 1-mediated proliferation, migration, and invasion of endometriotic cyst stromal cells. Reprod Biol Endocrinol. 17 (1): 69. DOI: 10.1186/s12958-019-0512-9 31443713 PMC6708153

[ref9] Koudelkova P., Costina V., Weber G., Dooley S., Findeisen P., Winter P., Agarwal R., Schlangen K., Mikulits W. (2017). Transforming Growth Factor-β Drives the Transendothelial Migration of Hepatocellular Carcinoma Cells. Int J Mol Sci. 18 (10): 2119. DOI: 10.3390/ijms18102119 PMC5666801 28994702

[ref10] Upagupta C., Shimbori C., Alsilmi R., Kolb M. (2018). Matrix abnormalities in pulmonary fibrosis. Eur Respir Rev. 27 (148): 180033. DOI: 10.1183/16000617.0033-2018 29950306 PMC9489108

[ref11] Iordanskaia T., Nawshad A. (2011). Mechanisms of transforming growth factor β induced cell cycle arrest in palate development. J Cell Physiol. 226 (5): 1415-24. DOI: 10.1002/jcp.22477 20945347 PMC3095042

[ref12] Murray J.C., Schutte B.C. (2004). Cleft palate: players, pathways, and pursuits. J Clin Invest. 113 (12): 1676-8. DOI: 10.1172/JCI22154 15199400 PMC420516

[ref13] Schutte B.C., Murray J.C. (1999). The many faces and factors of orofacial clefts. Hum Mol Genet. 8 (10): 1853-9. DOI: 10.1093/hmg/8.10.1853 10469837

[ref14] Heino J, Heinonen T. (1990). Interleukin-1 beta prevents the stimulatory effect of transforming growth factor-beta on collagen gene expression in human skin fibroblasts. Biochem J. 271 (3): 827-30. DOI: 10.1042/bj2710827 2244882 PMC1149639

[ref15] Kim J.H., Ham S, Lee Y., Suh G.Y., Lee Y.S. (2019). TTC3 contributes to TGF-β 1-induced epithelial-mesenchymal transition and myofibroblast differentiation, potentially through SMURF2 ubiquitylation and degradation. Cell Death Dis. 10 (2): 92. DOI: 10.1038/s41419-019-1308-8 30696809 PMC6351531

[ref16] Rajaram P., Chandra P., Ticku S., Pallavi B.K., Rudresh K.B., Mansabdar P. (2017). Epidermal growth factor receptor: Role in human cancer. Indian J Dent Res. 28 (6): 687-694. DOI: 10.4103/ijdr.IJDR_534_16 29256471

[ref17] Bogdan S., Klämbt C. (2001). Epidermal growth factor receptor signaling. Curr Biol. 11 (8): R292-5. DOI: 10.1016/s0960-9822(01)00167-1 11369216

[ref18] Zeng F., Harris R.C. (2014). Epidermal growth factor, from gene organization to bedside. Semin Cell Dev Biol. 28: 2-11. DOI: 10.1016/j.semcdb.2014.01.011 24513230 PMC4037350

[ref19] Miettinen P.J., Chin J.R., Shum L., Slavkin H.C., Shuler C.F., Derynck R., Werb Z. (1999). Epidermal growth factor receptor function is necessary for normal craniofacial development and palate closure. Nat Genet. 22 (1): 69-73. DOI: 10.1038/8773 10319864

[ref20] Li B., Ma L., Zhang C., Zhou Z., Yuan H., Jiang H., Pan Y., Tan Q. (2018). Associations of genetic variants in endocytic trafficking of epidermal growth factor receptor super pathway with risk of nonsyndromic cleft lip with or without cleft palate. Mol Genet Genomic Med. 6 (6): 1157-1167. DOI: 10.1002/mgg3.497 30411541 PMC6305670

[ref21] Wang X., Xu L., Lao Y., Zhang H., Xu H. (2018). Natural Products Targeting EGFR Signaling Pathways as Potential Anticancer Drugs. Curr Protein Pept Sci. 19 (4): 380-388. DOI: 10.2174/1389203718666170106104211 28059040

[ref22] Lindsey S., Langhans S.A. (2015). Epidermal growth factor signaling in transformed cells. Int Rev Cell Mol Biol. 314: 1-41. DOI: 10.1016/bs.ircmb.2014.10.001 25619714 PMC4888053

[ref23] Stefanini M., De Martino C., Zamboni L. (1967). Fixation of ejaculated spermatozoa for electron microscopy. Nature. 216 (5111): 173-4. DOI: 10.1038/216173a0 4862079

[ref24] Hsu S.M., Raine L., Fanger H. (1981). The use of antiavidin antibody and avidin-biotin-peroxidase complex in immunoperoxidase technics. Am J Clin Pathol. 75 (6): 816-21. DOI: 10.1093/ajcp/75.6.816 6167159

[ref25] Pilmane M., Luts A., Sundler F. (1995). Changes in neuroendocrine elements in bronchial mucosa in chronic lung disease in adults. Thorax. 50 (5): 551-4. DOI: 10.1136/thx.50.5.551 7541167 PMC1021228

[ref26] Chiang M.S., Yang J.R., Liao S.C., Hsu C.C., Hsu C.W., Yuan K. (2015). Latent transforming growth factor-β binding proteins (LTBP-1 and LTBP-2) and gingiva keratinization. Oral Dis. 21 (6):762-9. DOI: 10.1111/odi.12344 25858550

[ref27] Shin J.O., Lee J.M., Cho K.W., Kwak S., Kwon H.J., Lee M.J., Cho S.W., Kim K.S., Jung H.S. (2012). MiR-200b is involved in Tgf-β signaling to regulate mammalian palate development. Histochem Cell Biol. 137 (1): 67-78. DOI: 10.1007/s00418-011-0876-1 22072420

[ref28] Iwata J., Suzuki A., Yokota T., Ho T.V., Pelikan R., Urata M., Sanchez-Lara P.A., Chai Y. (2014). TGFβ regulates epithelial-mesenchymal interactions through WNT signaling activity to control muscle development in the soft palate. Development. 141 (4): 909–917. DOI: 10.1242/dev.103093 24496627 PMC3912833

[ref29] Raju G.T., Lakkakula B.V.K.S., Murthy J., Kannan M.A., Paul S.F.D. (2017). Transmission analysis of TGFB1 gene polymorphisms in non-syndromic cleft lip with or without cleft palate. Int J Pediatr Otorhinolaryngol. 100: 14-17. DOI: 10.1016/j.ijporl.2017.06.015 28802359

[ref30] Reutter H., Birnbaum S., Mende M., de Assis N.A., Hoffmann P., Lacava A.D., Herms S., Braumann B., Scheer M., Lauster C., Schmidt G., Schiefke F., Dunsche A., Martini M., Knapp M., Kramer F.J., Nöthen M.N., Mangold E. (2009). Transforming growth factor-beta receptor type 1 (TGFBR1) is not associated with non-syndromic cleft lip with or without cleft palate in patients of Central European descent. Int J Pediatr Otorhinolaryngol. 73(10): 1334-8. DOI: 10.1016/j.ijporl.2009.06.004 19586667

[ref31] Martinelli M., Scapoli L., Pezzetti F., Spinelli G., Lunardi S., Carinci F. (2009). Lack of association between common polymorphisms of epidermal growth factor receptors and nonsyndromic cleft lip with or without cleft palate. Int J Pediatr Otorhinolaryngol. 73 (7): 929-31. DOI: 10.1016/j.ijporl.2009.02.013 19307027

[ref32] Krivicka-Uzkurele B., Pilmane M., Akota I. (2008). Barx1, growth factors and apoptosis in facial tissue of children with clefts. Stomatologija. 10 (2): 62-6. https://sbdmj.lsmuni.lt/082/082-02.pdf 18708738

[ref33] Smane-Filipova L., Pilmane M., Akota I. (2016). Immunohistochemical analysis of nestin, CD34 and TGFβ3 in facial tissue of children with complete unilateral and bilateral cleft lip and palate. Stomatologija. 18 (3): 98-104. https://sbdmj.lsmuni.lt/163/163-05.pdf 28386053

[ref34] Krivicka B., Pilmane M., Akota I. (2013). Expression of growth factors and growth factor receptors in human cleft-affected tissue. Stomatologija. 15 (4): 111-8. https://sbdmj.lsmuni.lt/134/134-01.pdf 24589633

[ref35] Kouskoura T., Kozlova A., Alexiou M., Blumer S., Zouvelou V., Katsaros C., Chiquet M., Mitsiadis T.A., Graf D. (2013). The etiology of cleft palate formation in BMP7-deficient mice. PLoS One. 8 (3): e59463. DOI: https://doi.org/10.1371/journal.pone.0059463 23516636 10.1371/journal.pone.0059463 PMC3597594

[ref36] Smane-Filipova L., Pilmane M., Akota I. (2016). MMPs and TIMPs expression in facial tissue of children with cleft lip and palate. Biomed Pap Med Fac Univ Palacky Olomouc Czech Repub. 160 (4): 538-542. DOI: 10.5507/bp.2016.055 27876897

[ref37] Jankovska I., Pilmane M., Akota I. (2017). Expression of gene proteins, interleukins and β-defensin in cleft-affected tissue. Stomatologija. 19 (4): 103-108. https://sbdmj.lsmuni.lt/174/174-01.pdf 29806648

[ref38] Pilmane M., Sidhoma E., Akota I., Kazoka D. (2019). Characterization of Cytokines and Proliferation Marker Ki67 in Cleft Affected Lip Tissue. Medicina (Kaunas). 55 (9): 518. DOI: 10.3390/medicina55090518 PMC6780708 31443525

